# Antioxidants and Techno‐Functional Components of Squash (*Cucurbita moschata* L.) Pulp Powder From Tunisia

**DOI:** 10.1002/fsn3.70701

**Published:** 2025-08-05

**Authors:** Sonia Dhifli, Abderrahmen Chargui, Ichrak Kahri, Chedly Abidi, Mourad Jridi, Mondher Mejri

**Affiliations:** ^1^ Université de Jendouba, Ecole Supérieure d’Agriculture du Kef (ESAK), LR : Appui à la durabilité des systèmes de production agricoles du Nord‐Ouest Le Kef Tunisia; ^2^ Université de Jendouba, Institut supérieur de biotechnologie de Béja, Laboratoire de Physiologie Fonctionnelle et Valorisation des Bio‐Ressources (LPF‐VBR) Béja Tunisia

**Keywords:** antioxidants, fonctionnel food and processed product, phenolic compounds, squash pulp powder

## Abstract

Nutrition is a vital process, and the choice of food remains a constant concern for men to maintain a healthy life. Thus, with the evolution of alimentary habits and different nutritional resources, ensuring the quality of food has become a priority for humans to meet their nutritional needs and guarantee their well‐being. In this study, we highlighted the physicochemical characteristics and nutritional value of squash pulp powder as a processed product of good nutritional quality. In preliminary studies, powders dried differently were examined for proximate compositions (moisture, ash, total sugar, fat, protein, pigments, vitamin A and mineral) and techno‐functional properties. Besides its nutritional quality, pulp contains considerable amounts of total polyphenols, flavonoids, and tannins. Accordingly, it has interesting antioxidant power (IC50 = 1.667 μg/mL), indicating rapid and immediate antioxidant activity. These nutritional values and the presence of phenolic compounds qualify this squash pulp powder as a functional and conservable food product. Sensory analysis of biscuits made from 15% squash confirmed the nutraceutical characterization of squash pulp as a useful alimentary additive favoring the marketing of processed agricultural products. All the results emphasize the interest in using dried squash pulp in the food processing industry given its nutritional richness and easy cultivation.

## Graphical abstract

This study evaluates the nutritional profile and antioxidant potential of squash pulp powder. The powder, rich in phenolic compounds, vitamin A, and essential nutrients, demonstrates strong antioxidant activity (IC50 = 1.667 μg/mL). Techno‐functional analysis and sensory evaluation confirm its suitability as a functional food additive. Its incorporation into biscuits supports its valorization as a health‐promoting processed product.

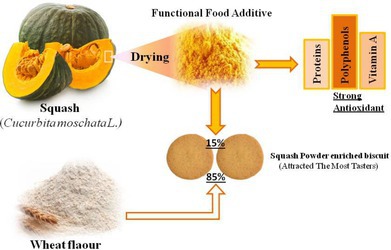

## 1 Introduction

In general, natural products, especially those of plant origin, are important sources of therapeutic agents. Currently, approximately a quarter of all drugs available for the treatment of diseases are derived from natural products (Atanasov et al. 2021). Indeed, plant products still represent an extremely valuable source for the production of new chemical entities for therapeutic purposes (Dzobo 2021). Thus, the valorization of plant resources food is a concern that is becoming increasingly important in many countries (Mondor et al. 2023). The significant progress made in the nutritional sciences, natural products, and functional foods has enabled better exploitation of plants or their processed natural substances with added value (Galanakis 2021). New concepts, such as nutraceuticals, nutritional therapy, phytonutrients, and phytotherapy, have thus been identified (Abdel‐Baki et al. 2022).

The nutraceutical, phytochemical, and pharmacological aspects of plants are largely linked to the presence of several substances, such as vitamins, tannins, flavonoids, carotenoids, and phenolic acids, which have strong antioxidant effects (Khatun et al. 2023; Boukid, Dall'Asta, et al. 2019). These compounds have attracted much interest in other fields, especially in the agri‐food industry, because of their ability to prevent various diseases (Rahman et al. 2021). Thus, these materials could constitute an alternative to synthetic food additives, which have shown harmful effects (Sambu et al. 2022). For this purpose, the concept of functional food is based on the ability to put on the market products that have desirable physiological effects greater than those that are usually associated with basic nutrients. As a general definition, a food is considered “functional” if it contains a dietary component that affects one or more specific functions of the body and has positive effects (*for review see* (Díaz et al. 2020)). Among the medicinal plants that constitute the plant cover are *Cucurbita moschata* , which is an annual herbaceous plant of the Cucurbitaceae family and the Cucurbita genus. There are three main domesticated species: *Cucurbita pepo* , *Cucurbita maxima* , and *C. moschata* . These plants are now cultivated on all continents. It is a traditional plant crop with great nutritional and economic importance that is cultivated from seeds and immature and mature fruits (Yalew 2023). Various studies have shown that squash is a good source of natural and effective antioxidants, such as polyphenols, vitamins, and carotenoids, which can be involved in maintaining good health thanks to their anti‐inflammatory, anticarcinogenic, and antimutagenic properties (Li 2020). Thus, the squash fruit has several physiological, immunological, pharmacological, and nutraceutical properties (Kaur et al. 2020). Among these fruits, butternut squash (*C. moschata dush*), which includes cucumber and tomato plants, is consumed as a vegetable, and this species is receiving increased interest due to its early maturity and attractive orange color to producers, traders, and consumers (Armesto et al. 2020).

Squash fruits are appreciated for their sweet taste, soft texture, low caloric content, and abundant nutritional value (Armesto et al. 2020). They are particularly valuable because of their richness in vitamins C, E, B6, K, thiamine, and riboflavin, as well as minerals such as potassium, phosphate, magnesium, iron, and selenium (Evranuz and Arduzlar‐Kağan 2015). Given their nutritional value and low production costs, squashes have great potential for use in industry. A range of products can be produced from squash, providing consumers with foods that have a long shelf life and good nutritional value (Mahmoud et al. 2022). In this context, the present work aimed to perform both physicochemical and techno‐functional characterization of dried squash powder ( *C. moschata* L.).

## 2 Materials and Methods

### 2.1 Plant Material

The plant material used in this study was squash pulp powder. Squash plants are a variety of *C. moschata* that are part of our plant heritage and eating habits. These species were chosen with the aim of developing this agroresource, which is of nutritional and commercial importance, into a functional food.

The squashes were washed, peeled, cut, and then crushed in an electric mixer grinder. The resulting paste was dried with three different drying modes: (1) Sun drying for 5 days, (2) Drying in an oven at 50°C until constant mass, and (3) Drying in a freeze dryer. The dry pulp was crushed and sifted again. The powders obtained were stored in sterile bottles. The latter were classified as clean or dry for 1 week before processing.

### 2.2 Nutritional and Functional Properties of Squash Powder

The physicochemical characteristics of the squash pulp powders were determined using techniques optimized in our laboratory. In fact, the fat content was determined in 5 g of the squash powder studied. The moisture and ash contents of the powder were evaluated using the gravimetric technique following the official method (Horwitz and Latimer 2000) with some modifications. The levels of organic matter and mineral salts in the squash pulp powder were determined in 1 g of sample following the AOAC method (In 1990).

### 2.3 Evaluation of Nutritional Properties

The moisture content was determined by gradual dehydration of the pulp in an oven until a zero mass variation was obtained (Weise et al. 1996). The total carbohydrate content was calculated as the difference between the dry weight and the weight of ash, protein, fat, and total fiber (Behera et al. 2009). The total lipid content was determined by continuous extraction in a Soxhlet apparatus for approximately 8 h using hexane as the solvent (Ramluckan et al. 2014). Total nitrogen was determined after mineralization of the samples using the Kjeldahl method with some modifications (Bremner 1960). The crude fiber content of the samples was determined by the Kűrschner‐Hanak method (Cvrk et al. 2022). The total ash and mineral contents were determined according to the methods of Pauwels and van Ranst (1992). Briefly, 5 g of squash powder from the sample was incinerated in a muffle furnace (Carbolite Eurotherm) at 550°C for 5 h, and the ash content was determined after cooling in a desiccator for 30 min. Minerals were extracted by successive digestion of 0.5 g of ash with nitric acid and per‐chlorohydric acid. The mixture was boiled for 10 min and filtered, after which the volume was increased to 100 mL with distilled water. The calcium content was determined with a flame photometer. Iron and zinc contents were evaluated by a UV–visible spectrophotometer. Each analysis was performed in quintuplicate.

### 2.4 Extraction and Quantification of Phenolic Compounds

The phenolic compounds in the squash pulp powder were extracted with absolute methanol or in water by simple maceration according to the method described by Turkmen et al. (2006) with some modifications. Extracts were prepared by adding 10 mL of the extraction solvent to 1 g of squash pulp powder. After stirring for 30 min, the mixture was kept at rest for 24 h at 4°C. The different extracts obtained were filtered using Wattman No. 4 paper (ashless filter) and stored at 4°C for subsequent analyses.

#### 2.4.1 Total Polyphenols

A total of 125 μL of the extract was diluted 10 times and mixed with 500 μL of distilled water and 125 μL of Folin–Ciocalteu reagent. After vigorous stirring of the mixture and 3 min of rest, 1250 μL of CO_3_ (Na) 2 at 7% was added. Finally, the mixture obtained was adjusted with distilled water to 3 mL. After a 90 min rest in the dark, the absorbance was read at a wavelength of 760 nm. The standard range was prepared with gallic acid at concentrations varying from 50 to 500 mg/L. Polyphenol contents are expressed in mg of gallic acid equivalent per gram of dry matter (mg EGA.g−1 DM).

#### 2.4.2 Total Flavonoids

A 0.25 mL dose of each extract was diluted fivefold and mixed with 0.075 mL of 5% sodium nitrite (NaNO_2_). The mixture was left to stand for 6 min before adding 0.15 mL of freshly prepared 10% aluminum chloride (AlCl_3_·6H_2_O). After a 5‐min incubation at room temperature, 0.5 mL of 1 M sodium hydroxide (NaOH) was added. The mixture was then adjusted with distilled water to a final volume of 2.5 mL. Absorbance was measured at 510 nm. A standard curve was prepared using catechin at concentrations ranging from 50 to 500 mg/L. Flavonoid content was expressed as milligrams of quercetin equivalent per gram of dry matter (mg QE/g DM).

#### 2.4.3 Total Tannins

Tannin concentrations were determined using the Folin–Ciocalteu method, with minor modifications (Ci and Indira 2016). Approximately 0.1 mL of the powder extract was mixed with 0.9 mL of H_2_O. Then, 0.5 mL of Folin–Ciocalteu reagent and 1 mL of 35% sodium carbonate solution were added. The mixture was diluted 10×, vortexed, and stored at room temperature for 30 min. At the same time, a standard range of tannic acid (20, 40, 60, 80, and 100 g/mL) was prepared. The absorbance was measured at 700 nm. The concentrations of TT in the squash pulp powder are expressed in mg equivalent tannic acid (ETA)/g DM.

#### 2.4.4 Condensed Tannins

Condensed tannins were determined using the vanillin method, with some modifications. To 1.25 μL of extract was added 1.5 mL of a 4% solution of vanillin in methanol and 75 μL of concentrated hydrochloric acid (HCl). After 30 min of incubation at 25°C, the absorption of the mixture was measured at 500 nm against methanol as a blank. Catechin was used as a standard for the calibration curve (0–0.2 mg/mL). The condensed tannin content of the different extracts was expressed in mg of equivalent catechin (EC)/g DM.

### 2.5 Carotenoid Content and Vitamin A

The procedure described by Leonardi et al. (2000) was followed with slight modification. Thirty milligrams of squash pulp powder were mixed with 1 mL of tetrahydrofuran (THF) in the presence of butylated hydroxytoluene (BHT) and resuspended in 5 mL of CHCl_3_. A further 1:10 dilution of the extracted material in 40% CH_3_CN, 20% methanol, 20% hexane, and 20% CH_2_Cl_2_ was performed before the chromatographic analysis. HPLC separation was carried out at a flow rate of 0.8 mL min−1 and a temperature of 30°C using a Chromaster 5160 HPLC with diode array detection and a Supelcosil C18 column (250 × 4.6 mm). Quantification was carried out by analyzing the chromatograms recorded at 450 nm for β‐carotene, zeaxanthin, and lutein; at 350 nm for phytofluene; and at 290 nm for phytoene. β‐carotene, zeaxanthin, phytoene, and phytofluene were quantified by calibration curves constructed with pure standards. Vitamin A was determined on the basis of β‐carotene and α‐carotene, which are provitamins A.

### 2.6 Antioxidant Assays: DPPH Radical Scavenging Activity

The antioxidant activity of the squash pulp powder was estimated using the DPPH (2,2 diphenyl‐1‐ picrylhydrazyl) radical scavenging method as described by Hashash et al. (2017), with slight modifications. The aliquots of different concentrations (0.1–4 mg/mL) of squash pulp powder (200 μL) were mixed with 125 μL of DPPH in methanol (0.2 mM) and deionized water (375 μL) and then incubated for 1 h in the dark. The positive standards (butylated hydroxytoluene and vitamin C) were prepared using the same procedure. The absorbance was measured at 517 nm. The scavenging activity of DPPH radicals was calculated according to the scavenging activity (%) = ((AbsControl − AbsSample)/AbsControl) × 100.

### 2.7 Techno‐Functional Properties of Squash Pulp Powder

#### 2.7.1 Determination of Water Holding Capacity

The water holding capacity (WHC) of a sample corresponds to the mass of water that it can absorb relative to its mass. WHC of orange fibers was determined by a centrifugation method according to Robertson et al. (2000) with the following modifications: 0.2 g of squash pulp powders were mixed in 3 mL of distilled water. The solution was mixed with a vortex mixer for 10 min and centrifuged at 12,000× *g* for 15 min, after which the supernatant was removed. The WHC was determined by the difference between the initial volume of the added water and the volume of the supernatant and was expressed in ml of water bound per g of powder.

#### 2.7.2 Determination of Oil Holding Capacity

The oil holding capacity (OHC) was determined according to the method described by Chen et al. (1988). Ten milligrams of powder was mixed with 1 mL of sunflower oil. The solution was mixed with a vortex mixer for 10 min and centrifuged at 12,000× *g* for 15 min, after which the supernatant was removed. The OHC was obtained by determining the difference between the initial volume of sunflower oil added and the volume of the supernatant and was expressed in mL of oil bound per g of powder.

#### 2.7.3 Determination of Emulsifying Properties

For each powder sample, the emulsifying activity and stability were determined at pH 2, 4, 6, and 8. Emulsifying activity is defined as the maximum amount of oil that can be emulsified by a fixed amount of the squash pulp powder, while the stability of the emulsion is defined as the rate of phase separation in water and oil during the storage of the emulsion (Pearce and Kinsella 1978). Powders were dissolved in distilled water at 60°C for 30 min, and the pH was adjusted with 1 M NaOH or 1 M HCl. Then, 30 mL of each solution at 2% and 3% was mixed with 10 mL of vegetable oil (i.e., soybean oil) for 1 min at room temperature and then homogenized using a Moulinex R62 homogenizer (IKA Werke GmbH & Co. Germany) at 16,000× rpm for 2 min. Two aliquots of each emulsion (50 μL) were pipetted at 0 and 10 min, diluted with 5 mL of a 0.1% sodium dodecyl sulfate (SDS) solution, and mixed thoroughly for 10 s using a vortex mixer. The absorbance was measured at 500 nm using a spectrophotometer (T70, UV/VIS spectrometer, PG Instruments Ltd., China). The emulsifying activity index (EAI) and the emulsion stability index (ESI) were calculated by Equations (2) and (3), respectively:

(1) $$\mathrm{EAI}\;\left(m^{2}/g\right)=\left(2\times 2.303\times A_{0}\times \mathrm{DF}\right)/\left(\left(1-\varphi \right)\times \mathrm{mp}\right)$$

(2) $$\mathrm{ESI}\;\left(\min\right)=\left(A_{0}\times \Delta t\right)/\left(A_{0}-A_{10}\right)$$

where *A*_0_ and *A*_10_ were the absorbance measured at initial time and after Δ*t* = 10 min, respectively. The variables *m*_*P*_ and φ stand for the mass of protein (g) and the volume fraction of oil in the emulsion, and DF is the dilution factor.

#### 2.7.4 Determination of Foaming Properties

Foam expansion (FE) and foam stability (FS) of the squash pulp powder solutions were determined according to the methods of Shahidi et al. (1995), with slight modifications. Each powder solution was prepared at 2% and 3% concentration and homogenized at high speed of 15,000 rpm for 1 min. The mixture was carefully transferred into a 50 mL cylinder for volume measurement. FE, which characterizes the foaming capacity of squash pulp powder solution, was measured by optical devices attached to the FoamScan system during the foaming process. FE was calculated by the following equation:

$$\mathrm{FE}\left(\%\right)=\left(\left(V1-V0\right)/V0\right)\times 100;$$

where *V*_0_ is volume of foaming solution at the start and *V*_1_ is volume of foaming solution at the end.

#### 2.7.5 Determination of Solubility of Squash Pulp Powder

The determination of the solubility of the squash pulp powder was carried out according to the methods described by Goula et al. (2004), with some modifications. One gram of powder was dissolved in 50 mL distilled water, and then the mixture was agitated with a magnetic stirrer at position 5. The resulting suspension was heated to 80°C for 30 min in a water bath. The mixture was cooled to 30°C ± 2°C and centrifuged at 7800 rpm for 15 min. The supernatant was subjected to high temperature until drying. The residue obtained after drying the supernatant represents the quantity of powder solubilized in water. The solubility was calculated in g per 100 g of powder based on the dry weight.

#### 2.7.6 Determination of the Minimum Gelation Concentration

The minimum gelation concentration of each sample was carried out by the method described by Sun and Arntfield (2010), with some modifications. Squash pulp powders were dissolved in distilled water at different concentrations (2%–10%). Two milliliters of each prepared dispersion was transferred to a test tube. The tubes were heated in a boiling water bath for 1 h, followed by rapid cooling in a cold water bath. The test tubes were then cooled to 4°C for 2 h. The least gelation concentration was determined by the method given as that concentration when the sample from the inverted tube did not slip.

### 2.8 Biscuit Formulation Test

The formulation test was carried out with the squash powder, which had the highest nutrient content. The powder was used to replace flour in several proportions: 0%, 5%, 10%, and 15%. The mixture was homogenized, and ingredients such as sugar, flour, egg, vanilla sugar, butter, salt, yeast, and freeze‐dried powder were obtained. The mixture was homogenized again to obtain the dough, which was wrapped in aluminum foil and kept in the refrigerator for 1 h. The sample was then rolled on a cutting board to a uniform thickness of approximately 5 mm. The dough was cut into 4 cm circular pieces, placed on a perforated tray, transferred to an oven preheated to 180°C, and baked for 10 min. The shortbreads were removed from the oven, cooled to room temperature, and stored in tightly closed packages for the evaluation test.

### 2.9 Sensory Evaluation of Biscuits

The biscuits were submitted to a panel of 250 tasters who were invited to analyze the products and complete the sensory evaluation sheets for the cooked biscuit. The following four criteria were evaluated: color, odor, taste, hardness, and overall appearance. A score between 1 and 5 was assigned to each criterion and each sample studied. Each tasting was followed by gargling with water to avoid any aroma interference.

### 2.10 Statistical Analysis

The results are presented as the means ± standard errors. Analysis of variance (ANOVA) was carried out using IBM SPSS Statistics software version 20, and the data are presented in the form of tables and histograms. For all the statistical analyses, the significance threshold was set at *p* < 0.05 (*), *p* < 0.01 (**).

## 3 Results

### 3.1 Physicochemical Composition of *C. moschata* Squash Pulp Powder

The results of biochemical analyses of dried squash pulp powders are of great interest for evaluating their nutritional potential. Table 1 presents the moisture, ash, sugar, lipid, and protein contents of the squash pulp powders obtained by three drying methods: solar drying, oven drying, and freeze drying (lyophilization).

**TABLE 1:** Water content and macronutrient composition of the squash pulp powders.

Drying mode	Humidity (%)	Ash (%)	Total sugar (%)	(%) Protein	(%) Lipid
Solar drying	7.65 ± 0.88^c^	5.26 ± 0.4^a^	74.89 ± 1.84^a^	7.8 ± 0.2^a^	3.2 ± 0.27^b^
Oven drying	8.52 ± 0.17^b^	4.13 ± 0.2^b^	72.68 ± 1.25^a^	8.4 ± 0.4^a^	3.59 ± 0.24^b^
Lyophilization	9.68 ± 0.42^a^	5.07 ± 0.3^a^	73.64 ± 0.99^a^	8.5 ± 0.4^a^	4.01 ± 0.23^a^

*Note:* Means labeled with different letters are considered statistically different at the 5% probability level.

### 3.2 Moisture of Powders Dried Differently

Moisture content different drying methods show significant effects on the powder's moisture properties (*p* < 0.05). The freeze‐dried squash pulp powder had the highest moisture content (9.68%), followed by oven drying (8.52%) and solar dried squash pulp powder (7.65%). The moisture content of squash pulp powder is comparable to those reported by Lim et al. (2021). Indeed, water content > 12% in flour or powder promotes the development of microorganisms. In our study, the water content was < 10%. For all powders dried differently, this process could provide good results during storage.

### 3.3 Ashes of Powders Dried Differently

The ash contents ranged between 5.26% and 5.07%; these values are lower than those shown by Lim et al. (2021), but much greater than those of sweet potato flour described by Chancelle Betty Ndangui (2015). The latter described values on the order of 1.2% ± 0.1%. From these results, we can deduce that the squash and pumpkin pulp powders are rich in ash, and these contents are related to the mineral composition.

#### 3.3.1 Total Sugar Content of Powders Dried Differently

The total sugar contents of squash pulp powder varied between 72.68% and 74.89%; these values are comparable to those shown in pumpkin powder (Lim et al. 2021). However, in sweet potato flour, the carbohydrate content exceeds 85% (Ndangui 2015). This indicates squash is a calorie bargain.

### 3.4 Protein Content of Powders Dried Differently

The protein content of the three powders studied varied between 7.8% and 8.5%. These amounts of protein are comparable to those found in pumpkin powder (Lim et al. 2021). But these protein content values are much greater than those of sweet potato flour described by Chancelle Betty Ndangui (2015). The results showed that squash and pumpkin pulp powders are richer in protein than sweet potato flour.

### 3.5 Lipid Content of Powders Dried Differently

The lipid contents of squash pulp powders range between 3.2% and 4.01% for the three powders dried differently. These values are also comparable to the fat contents shown in pumpkin powder (Lim et al. 2021). However, squash pulp powder has a higher lipid content than that determined in sweet potato, which does not exceed 1%.

### 3.6 Contents of Vitamin A and Carotenoid Pigments of Powders Dried Differently

The analysis of the powders studied showed significant values of carotenoids, including β‐carotene, which was the major element: 120 μg/g (Table 2). In descending order, lutein had a value of 1.012 μg/g, followed by zeaxanthin with a value of approximately 0.11 μg/g, phytoene with a value of 0.04 μg/g, and phytofluene with 0.03 μg/g. The vitamin A indices for the three powders ranged from 210 to 251 μg/100 g. The powders studied had high levels of vitamin A compared to those of marketed flours containing hard wheat flour, which was low in the latter. The recommended daily dose of vitamin A for an adult is approximately 600 μg according to the World Health Organization (1982). The results showed that 100 g of squash powder could provide half of the recommended daily dose of vitamin A, especially in powders undergoing solar drying, which show high levels of vitamin A and lutein.

**TABLE 2:** Levels of carotenoids and vitamin A in squash pulp powder (μg/g).

Drying mode	Lutein (μg/g)	Zeaxanthin (μg/g)	Phytoene (μg/g)	Phytofluene (μg/g)	β‐carotene (μg/g)	Vitamin A (μg/100 g)
Solar drying	1.012^a^	0.11^a^	0.03^a^	0.02^a^	120^a^	251 ± 1.25^a^
Oven drying	0.658^c^	0.10^a^	0.04^a^	0.03^a^	114^b^	249 ± 2.61^a^
Lyoliphization	0.971^b^	0.09^a^	0.01^a^	0.02^a^	116^b^	210 ± 0.14^c^

*Note:* Means labeled with different letters are considered statistically different at the 5% probability level.

### 3.7 Determination of Metal Ions

The mineral salt profile presented in Table 3 indicates that calcium was the major element, with 1054 ppm in solar drying squash pulp powder, which presents 68.31 ppm of iron and 44.89 ppm of zinc. These values are much greater than those of sweet potato flour described by Chancelle Betty Ndangui (2015).

**TABLE 3:** Mineral composition of the squash pulp powders.

Drying mode	Zn (ppm)	Fe (ppm)	Ca (ppm)
Solar drying	44.89 ± 2.69^a^	68.31 ± 3.61^a^	1054 ± 10.75^a^
Oven drying	42.4 ± 1.0^b^	52.12 ± 6.69^a^	1005.78 ± 8.79^b^
Lyoliphization	43.07 ± 0.21^c^	71.2 ± 5.19^a^	998.78 ± 6.91^c^

*Note:* Means labeled with different letters are considered statistically different at the 5% probability level.

The enrichment of the diet with essential nutrients (sugars, proteins, lipids), as well as the quantities of mineral salts, carotenoids and vitamin A present in squash pulp powder have clearly shown the potential for using these powders in functional and dietetic foods.

### 3.8 Techno‐Functional Properties of *C. maxima* Squash Powder

The functional properties (WHC, OHC, solubility, emulsification, foaming power and gelling abilities) are intrinsic physicochemical characteristics of powders that can be affected during the processing and storage of food products. The functional properties of these materials provide information on the potential applications of powders in food formulation (Hermansson 1979). Product development is generally based on the functional properties that we wish to obtain. The functionality of ingredients and additives is highly important and is the focus of producers, buyers, and consumers.

In Table 4, we show the techno‐functional properties of squash pulp powders by studying these characteristics with three methods of drying: solar drying, oven drying, and lyophilization.

**TABLE 4:** Techno‐functional properties of squash pulp powder.

Drying mode	WHC (g of water/g DS)	OHC (g of oil/g DS)	Soluble solids content (g/100 g)	Concentartion	Emulsion activity index (m^2^/g)	Foaming power (%)
Solar drying	5.97 ± 0.1^a^	4.52 ± 0.12^a^	6.1 ± 0.0^b^	2% 3%	24.68 ± 1.1^b^ 52.91 ± 1.5^a^	67.78 ± 1.24^b^ 103.65 ± 2.68^a^
Oven drying	4.12 ± 0.1^b^	3.67 ± 0.10^b^	5.7 ± 0.1^b^	2% 3%	20.14 ± 2.01^b^ 53.79 ± 1.97^a^	58.13 ± 1.66^b^ 99.75 ± 3.17^a^
Lyophilization	3.68 ± 0.14^c^	2.94 ± 0.09^c^	7.4 ± 0.02^a^	2% 3%	20.94 ± 3.1^b^ 42.30 ± 2.43^a^	40.34 ± 0.59^b^ 82.33 ± 4.68^a^

*Note:* Means labeled with different letters are considered statistically different at the 5% probability level.

#### 3.8.1 Water Holding Capacity (WHC) and Oil Holding Capacity (OHC)

Water holding capacity (WRC) and oil holding capacity (OHC) of squash pulp powders indicated in Table 4 were significantly (*p* < 0.05) different for the three drying conditions. The water retention capacity was on the order of 5.97 for solar dried powder, 4.12 for oven dried powder, and 3.68 g/g DS of lyophilized powder. These values correspond to the mass of water in grams absorbed relative to the weight of the sample and characterize the content of hydrophilic compounds in the squash pulp powder, which are sensitive to the way of drying. Water is an essential participant in the stability, structure, dynamics, and function of proteins and other biomolecules. However, the protein conformation and environmental parameters can change its structure and affect the amount of water absorbed (Nikbakht Nasrabadi et al. 2021). Moreover, other molecules, such as carbohydrates and ions, in the powder can also influence this hydration property (Roy et al. 2016). Indeed, adding an ingredient with a high water absorption capacity can improve the texture of a product. This approach also helps improve the roughness of meat products after cooking. The OHCs of the powders were 4.52, 3.67, and 2.94 g oil/g DS for solar drying, oven drying, and freeze drying, respectively. These are directly related to the proportion of hydrophobic compounds in the squash pulp powder, which are also sensitive to the way of drying. Several studies have reported that hydrophobic constituents, such as insoluble fibers, are the main reason for OHC (Biswas et al. 2011; Bouaziz et al. 2020). This property can be exploited in certain foods to improve fat and flavor retention and increase feed yield.

The WHC and OHC of *C. moschata* squash powders are very close to those of the marketed ingredients soy and pea protein isolates, gum arabic, beet pectin, and corn fiber (Kamga 2019). Therefore, squash pulp powder can be exploited as a texturing agent in food preparations.

#### 3.8.2 Soluble Solids Content

As shows in Table 4, the solubility of squash pulp powder is significantly high with lyophilized powder (7.4 g/100 g) than solar dried (6.1 g/100 g) and oven dried (5.7 g/100 g) powders. This reflects the conservation of other molecules of squash pulp powder in drying by lyophilization. But this value is still lower than the soluble solid content shown in fresh pumpkin pulp (8.97 g/100 g) (Ghendov‐Mosanu et al. 2023).

#### 3.8.3 Emulsifying Property

Emulation is a dispersion of a liquid phase in the form of droplets in another miscible liquid phase called dispersing. It is defined by three phenomena: (i) diffusion, (ii) attachment of adsorbed protein molecules, and (iii) rearrangement of adsorbed molecules at the interface can occur (Panyam and Kilara 1996). The most commonly studied parameter for characterizing emulsions is emulsifying capacity (grams of emulsified oil per gram of protein). In Table 4, we show the emulsifying properties and the stability of the squash pulp powder emulsions. Our results show no significant difference between the three drying methods. But when the concentration was increased from 2% to 3%, the emulsification power doubled for the three powders studied (Table 4).

#### 3.8.4 Foaming Power

The foaming power shows no significant difference between the three powders studied, which present 67.78% for solar dried powder, 58.13% for oven dried powder, and 40.34% of lyophilized powder. But by increasing the concentration from 2% to 3%, we observed a doubling of the foaming power values (Table 4).

#### 3.8.5 Gelling Property

Measuring the minimum gelling concentration allows determination of the conditions under which a protein is capable of gelling. Gelling proteins are used to impart texture to products or as a binder. In our study, the gelling test for the three powders studied showed no gelling power, which indicates the low amount of protein in squash pulp powder.

### 3.9 Phenolic Compound Content

**FIGURE 1:**
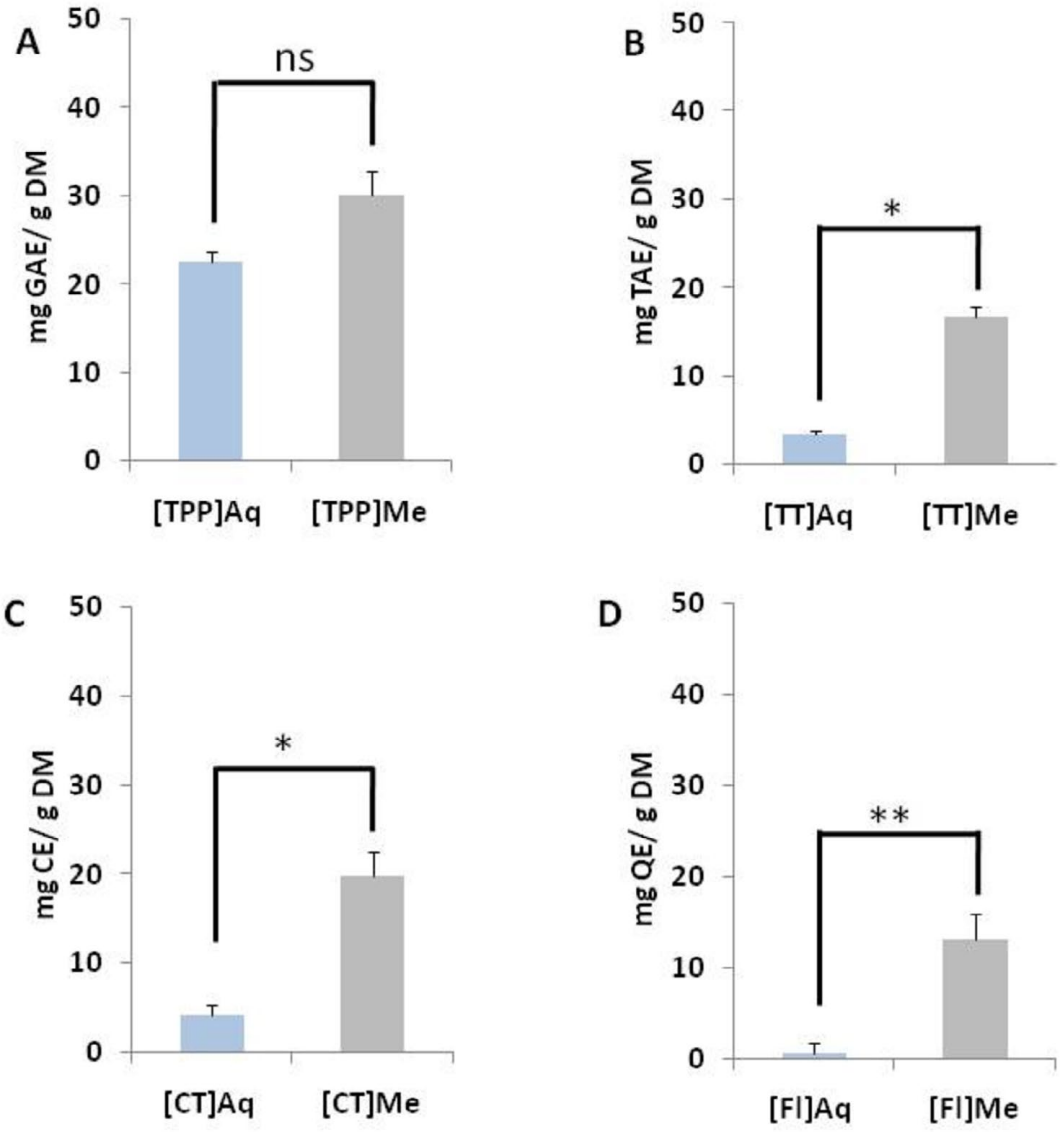
Polyphenolic Content of Squash Pulp Powder (Cucurbita moschata L.) Extracts. (A) Total polyphenols, (B) total tannins, (C) condensed tannins, and (D) flavonoids contents extracted from squash pulp powder ( *C. moschata* L.) in aquanious or methanol media. The data are presented as mean values ± standard error (*n* = 5). Values are expressed as CE, catechin equivalent; GAE, gallic acid equivalent; QE, quercetin equivalent; TAE, tanic acid equivalent. Statistical significance is indicated by *p < 0.05 and **p < 0.01.

Phenolic compounds were measured in freeze‐dried squash pulp powder. Figure 1 shows comparison histograms between aqueous extraction and methanol extraction of different phenolic compounds such as total polyphenol, total and condensed tannin, and flavonoid. Previous studies have found that the content of phenolic compounds per 100 g of fresh weight for some types of *C. moschata* varies between 26 and 80 mg (Priori et al. 2016; Kulczyński and Gramza‐Michałowska 2019).

#### 3.9.1 Total Polyphenol Content

Total polyphenol (TPP) content of the squash pulp was shown in Figure 1A. The comparison of TTP histograms between aqueous extraction (22.496 ± 1.159 mg GAE/g DM) and methanol extraction (30.103 ± 2.830 mg GAE/g DM) indicates no significant difference in the level of TPP between the two extraction methods.

#### 3.9.2 Total Tannin Content

The histograms in Figure 1B show a comparison of the total tannin (TT) between aqueous extraction (3.541 ± 1.004 mg TAE/g DM) and methanol extraction (16.792 ± 0.364 mg TAE/g DM). This indicated a significant difference (*p* < 0.05) between the two extraction methods (Figure 1B). Using different solvents of extraction, Ethiraj Sumathi and Balasundaram Janarthanam show that tannin concentration in squash seed was between 14 and 20 mg TAE/g DM (Ethiraj and Balasundaram 2016).

#### 3.9.3 Condensed Tannin Content

Figure 1C illustrates a significative difference (*p* < 0.05) in the content of condensed tannin in squash pulp powder between aqueous extraction and methanol extraction. Indeed, the extraction of condensed tannin compounds was significantly affected by the extraction solvent (Enneb et al. 2020). The quantification of condensed tannins in the extracts revealed that the highest quantity was in the methanol extract (20,608 ± 2035 mg CE/g DM), while the aqueous extracts had a content of approximately 4.215 ± 0.833 mg CE/g DM (Figure 1C).

#### 3.9.4 Flavonoids Content

Flavonoids content was determined using the standard range of quercetin which was read at 510 nm. Figure 1D shows the results of the extraction of flavonoids from the powder of the squash pulp; the statistical analysis revealed significant differences (*p* < 0.01) depending on the extraction method. The histograms above (Figure 1D) indicate that the highest content of flavonoids is obtained in the methanol extract (13 ± 0.612 mg QE/g DM), while the aqueous extract was 0.561 ± 0.060 mg QE/g DM. Using different extraction media, Enneb et al. (2020) show that methanol was the finest extraction solvent compared to hexane, chloroform, and ethyl acetate.

### 3.10 Antioxidant Activity of Squash Pulp Powder

**FIGURE 2:**
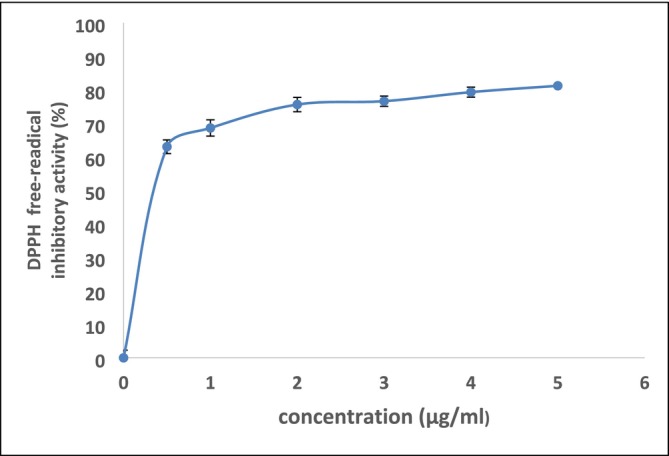
DPPH free radical scavenging activity by methanolic extract of squash pulp powder DPPH free‐radical inhibitory activity (%) of methanolic squash pulp powder extract ( *C. moschata* L.) at various concentrations (0–5 μg/mL). The graph demonstrates a concentration‐dependent increase in antioxidant activity, with a sharp rise at lower concentrations and a plateau at higher concentrations, indicating saturation of radical scavenging capacity. Data are presented as mean ± standard deviation (*n* = 5).

This activity was determined by trapping the free radical DPPH via the antioxidant molecules present in the squash pulp powder. Figure 2 shows the evolution of free radical scavenging activity in the methanol extracts.

In fact, the DPPH radical trapping activity of squash pulp powders is immediate. This is also explained by the IC50 value (1.667 μg/mL), which is very low. Indeed, the use of freeze‐dried squash pulp powder is a good preservation method that ensures the stability of antioxidant substances and phenolic compounds. Thus, squash pulps can be marketed as food products in the form of powders for the preparation of components, yogurt, soup, etc. (Dari 2013; Delgado‐Nieblas et al. 2017), or mixed with other food products such as biscuits (López‐Mejía et al. 2019).

In addition, the considerable presence of phenolic compounds in the powder as well as mineral salts, organic matter, especially sugars, and vitamins makes squash pulp powder a processed product of good food quality that can be qualified as a functional food. Thus, a sensory analysis of this powder was carried out to confirm the nutritional and functional characteristics of the pulp of *C. moschata* squash as an additive, enriching the sensory value of marketed food products.

### 3.11 Sensory Analysis of Biscuit

Sensory analysis consists of studying the properties of a product in an ordered and structured manner to be able to describe it, classify it, or improve it in an extremely objective and rigorous way (Ruiz‐Capillas and Herrero 2021). The examination of the organoleptic properties (texture, flavor, taste, appearance, smell, etc.) of the biscuit is carried out through the senses (sight, smell, taste, touch and hearing) of the panelists (Table 5).

**TABLE 5:** Results of sensory tests.

	Control	5%	10%	15%
Color	3.15 ± 0.86	3.26 ± 1.07	2.95 ± 0.99	3.98 ± 1.05
Smell	3.6 ± 1.08	2.63 ± 1.10	3.15 ± 0.98	3.98 ± 0.92
Taste	3.30 ± 1.09	3.05 ± 0.96	2.55 ± 0.88	3.73 ± 1.11
Hardness	3.20 ± 0.76	2.50 ± 0.96	3.55 ± 0.75	2.25 ± 0.95
Overall appearance	3.48 ± 0.99	3.55 ± 1.15	3.20 ± 1.18	3.83 ± 1.22

**FIGURE 3:**
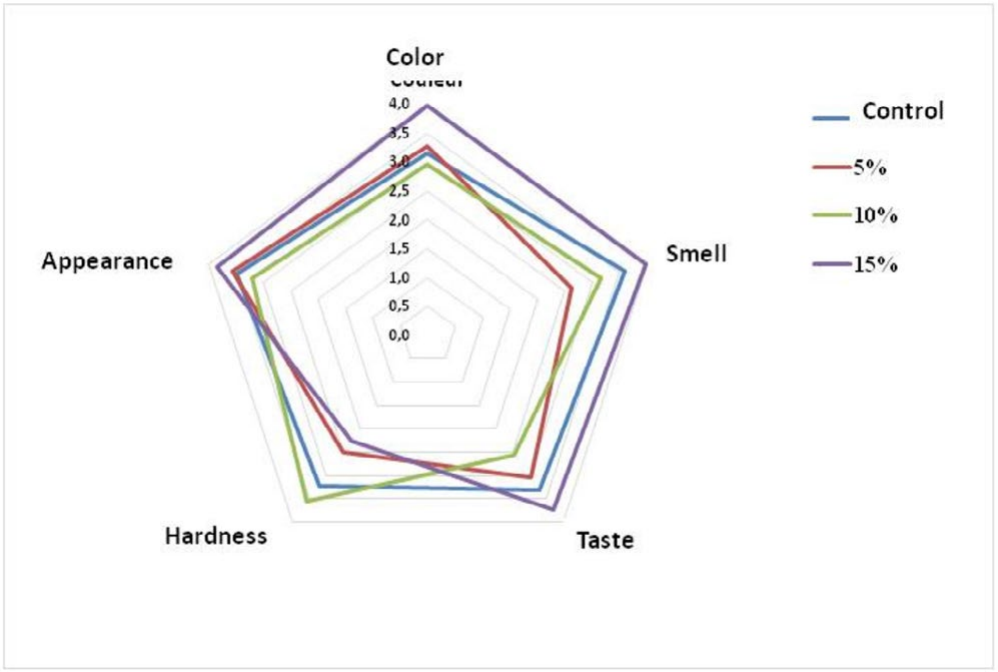
Radar Diagram of Sensory Evaluation of Squash Pulp Powder‐Enriched Samples (Cucurbita moschata L.). Radar diagram representing the sensory evaluation of samples enriched with varying concentrations (0%, 5%, 10%, and 15%) of squash pulp powder. Sensory attributes assessed include color, smell, taste, hardness, and appearance. Panelists scored each attribute using a 5‐point hedonic scale, with higher scores indicating greater acceptability. The control sample (0%) is represented in blue, while the enriched samples are shown in red (5%), green (10%), and purple (15%).

According to this presentation (Figure 3), the best scores for color (3.98), taste (3.73) and smell (3.98) were recorded for biscuits made from 15% squash and 85% flour. These results are superior to those of the control biscuits, which were 3.15 of color, 3.30 of taste and 3.60% of smell, and to those of the biscuits based on 5% and 10% squash pulp (Table 5). Thus, by reducing the quantity of squash powder added to the flour, we noticed that the main sensory characteristics of the biscuits decreased, which was favorable for the consumer.

In addition, the 15% squash pulp powder biscuits had the best overall appearance score (3.83) compared to the 5% (3.20), control (3.48), and 10% (3.55%) biscuits. This also shows that adding a considerable quantity of pulp to flour to produce biscuits increases the factor that attracts the consumer. These results illustrate that the biscuits enriched with 15% squash pulp powder were much more colorful than the control biscuits and had a positive influence on the acceptability of the product. Indeed, color seems to be a very important criterion for the initial acceptability of biscuits by consumers.

## 4 Discussion

The water content of the squash pulp powder ranged between 10% and 12% aligning with the FAO guidelines, which recommend that the moisture content of dried vegetables should be below 12% (Food and Agriculture Organization of the United Nations 1995). This suggests that the drying methods used were effective in preserving the product within safe moisture levels. The lipid content of the *C. moschata* squash powder samples was low, consistent with previous findings by Wongsagonsup et al. (2015). These low lipid values indicate the suitability of squash powder as a fat‐reducing ingredient in high‐fat meals and its potential inclusion in weight management diets, as discussed by Dzobo (2021) in the context of plant‐based health products.

The total sugar contents of the samples ranged from 73% to 75%, aligning with the findings of Adelaïde et al. (2021) and indicating consistent sweetness levels across samples. The dietary fibers content in the squash powder further underscores its health benefits. As an indigestible component, fibers support proper intestinal function by increasing stool bulk and reducing gastrointestinal transit time. A fiber‐deficient diet has been linked to several health conditions, including heart disease, colon and rectal cancer, varicose veins, obesity, appendicitis, diabetes, and constipation (Atanasov et al. 2021; O'Keefe 2019).

Micronutrient and carotenoid content were varied depending on the drying method used. Notably, solar‐dried samples showed higher carotenoid levels than those dried using oven or lyophilization methods, although the differences were not statistically significant. Carotenoids and polyphenols are widely known for their antioxidant and health‐promoting properties (Rahman et al. 2021; Fernandes et al. 2019). Ash and mineral contents—including zinc, iron, and calcium—were similarly high across all samples. These minerals play essential roles in physiological functions such as bone and teeth formation (calcium), hemoglobin synthesis (iron), and immune system support (zinc) (*for review see* (Boukid, Zannini, et al. 2019)).

The squash pulp powders were also found to be low in energy, making them suitable for individuals with low to moderate physical activity levels (Adelaïde et al. 2021). However, to ensure a balanced diet and facilitate the absorption of provitamin A, they should be consumed alongside high‐fat foods. In addition to vitamin A, the powders contained significant levels of phenolic compounds—including polyphenols, tannins, and flavonoids—as well as vitamin C, indicating strong antioxidant activity (Galanakis 2021; Atanasov et al. 2021; Adelaïde et al. 2021).

Functionally, squash pulp powder demonstrated high swelling capacity, solubility, oil absorption, and water retention, making it useful as a thickener in liquid and semi‐liquid food processing (Barber et al. 2020; Noor Aziah and Komathi 2009). Variations in functional and nutritional properties reported across studies can be attributed to differences in geographical location, climatic conditions, soil type, plant maturity, and genetic makeup (*for review see* (Gimah et al. 2025)). Additional factors such as treatment conditions, composition, grain size, density, pH, and storage conditions also influence the water solubility of powdered products (Barber et al. 2020).

A sensory evaluation of biscuits made with squash pulp powder and wheat flour revealed that samples containing 15% squash pulp powder and 85% wheat flour were the most preferred by panelists, even surpassing the control biscuits without squash pulp powder. These biscuits also had a more appealing color, which positively influenced their overall acceptability. Similar results were reported by Adelaïde et al. (2021) and (Saadoudi et al. 2024), who found that substitution levels of 10%–15% squash pulp powder improved taste and antioxidant activity. These findings are consistent with the growing interest in functional bakery products enriched with bioactive ingredients (Abdel‐Baki et al. 2022). In our study, all three samples were well received by consumers, but type C (15% squash pulp, 85% flour) attracted the most tasters.

Finally, water activity and pH were identified as critical factors in preserving squash‐based products, as improper control could lead to microbial contamination. Therefore, stability control and microbiological testing are essential during product development and commercialization (Sambu et al. 2022).

## 5 Conclusion

In summary, squash pulp powder is a nutritionally rich, low‐energy food with promising health benefits due to its high fiber, mineral, vitamin, and antioxidant content. Its functional properties also support its use in food formulation, particularly as a thickening agent. When used in baked goods, a 15% substitution rate strikes a balance between nutritional enhancement and sensory acceptability. However, attention must be paid to microbial stability during processing and storage. These findings highlight the potential of squash pulp powder as a valuable ingredient in health‐oriented and functional food products.

## Author Contributions

**Sonia Dhifli:** conceptualization (equal), data curation (equal), investigation (equal), methodology (equal), writing – original draft (equal). **Abderrahmen Chargui:** conceptualization (equal), methodology (equal), project administration (equal), resources (equal), supervision (equal), validation (equal), writing – original draft (equal), writing – review and editing (equal). **Ichrak Kahri:** data curation (equal), investigation (equal), methodology (equal), writing – original draft (equal). **Chedly Abidi:** data curation (equal), investigation (equal), validation (equal). **Mourad Jridi:** funding acquisition (equal), project administration (equal), resources (equal), validation (equal), writing – review and editing (equal). **Mondher Mejri:** conceptualization (equal), funding acquisition (equal), project administration (equal), resources (equal), supervision (equal), validation (equal), writing – review and editing (equal).

## Conflicts of Interest

The authors declare no conflicts of interest.

## Data Availability

The data that support the findings of this study are available on request from the corresponding author. The data are not publicly available due to privacy or ethical restrictions.
